# AUCReshaping: improved sensitivity at high-specificity

**DOI:** 10.1038/s41598-023-48482-x

**Published:** 2023-11-30

**Authors:** Sheethal Bhat, Awais Mansoor, Bogdan Georgescu, Adarsh B. Panambur, Florin C. Ghesu, Saahil Islam, Kai Packhäuser, Dalia Rodríguez-Salas, Sasa Grbic, Andreas Maier

**Affiliations:** 1 Pattern Recognition Lab, Friedrich-Alexander-Universität Erlangen-Nürnberg, 91058 Erlangen, Germany; 2 Digital Technology and Innovation, Siemens Healthineers, Erlangen, Germany; 3 Digital Technology and Innovation, Siemens Medical Solutions, Princeton, NJ 08540 USA

**Keywords:** Engineering, Electrical and electronic engineering

## Abstract

The evaluation of deep-learning (DL) systems typically relies on the Area under the Receiver-Operating-Curve (AU-ROC) as a performance metric. However, AU-ROC, in its holistic form, does not sufficiently consider performance within specific ranges of sensitivity and specificity, which are critical for the intended operational context of the system. Consequently, two systems with identical AU-ROC values can exhibit significantly divergent real-world performance. This issue is particularly pronounced in the context of anomaly detection tasks, a commonly employed application of DL systems across various research domains, including medical imaging, industrial automation, manufacturing, cyber security, fraud detection, and drug research, among others. The challenge arises from the heavy class imbalance in training datasets, with the abnormality class often incurring a considerably higher misclassification cost compared to the normal class. Traditional DL systems address this by adjusting the weighting of the cost function or optimizing for specific points along the ROC curve. While these approaches yield reasonable results in many cases, they do not actively seek to maximize performance for the desired operating point. In this study, we introduce a novel technique known as AUCReshaping, designed to reshape the ROC curve exclusively within the specified sensitivity and specificity range, by optimizing sensitivity at a predetermined specificity level. This reshaping is achieved through an adaptive and iterative boosting mechanism that allows the network to focus on pertinent samples during the learning process. We primarily investigated the impact of AUCReshaping in the context of abnormality detection tasks, specifically in Chest X-Ray (CXR) analysis, followed by breast mammogram and credit card fraud detection tasks. The results reveal a substantial improvement, ranging from 2 to 40%, in sensitivity at high-specificity levels for binary classification tasks.

## Introduction

In recent years, Deep Learning (DL) systems have gained prominence in a wide array of domains, including medical imaging, industrial automation, manufacturing, cyber security, fraud detection, drug research, and insurance, among others^1–6^. The prevailing approach for assessing DL system performance is the calculation of the Area Under the Receiver Operator Curve (ROC), denoted as the AUC score. Nonetheless, the AUC score does not account for the potential costs of misclassifications, neglecting the different implications of false positives and false negatives^7^. This limitation may render it less suitable for situations in which the costs associated with specific types of errors significantly differ, owing to the overarching nature of this metric.

Many of these applications frequently involve datasets with class imbalances, as seen in cases such as credit card fraud detection^8^ or intruder detection from doorbell cameras^9^. The AUC score, as a metric, exhibits insensitivity to class imbalances and a-priori probabilities. To address class imbalance, the conventional approach involves techniques like undersampling, oversampling, threshold adjustment, or the introduction of varying costs within the loss function to penalize misclassifications^10,11^. Subsequently, an operating point is chosen on the ROC curve to achieve the desired levels of specificity and sensitivity. Nevertheless, this approach may disregard a-priori probabilities, resulting in significant variations in the performance of most deployed DL models^12,13^.

Moreover, it is a prevailing practice to design DL systems that necessitate exceptional performance under specific conditions, such as high sensitivity or specificity, making the performance across the remainder of the ROC curve of lesser significance. While a system may exhibit a high AUC score, its suitability in a real-world context can be uncertain. This is because the cost functions employed to optimize the DL network are oriented toward holistic optimization of the entire ROC curve, potentially resulting in sub-optimal performance for the deployed system^12,13^. Such a system, runs at specific values of false-positive rate and sensitivity which corresponds to a single point on the ROC curve. The high AUC score achieved by the system does not necessarily indicate the desired performance or minimal misclassifications of one class at the chosen operating point. It is, therefore, customary to look at the shape of the curve, rather than merely going with the AUC scores when selecting the best model for deployment.

**Figure 1:**
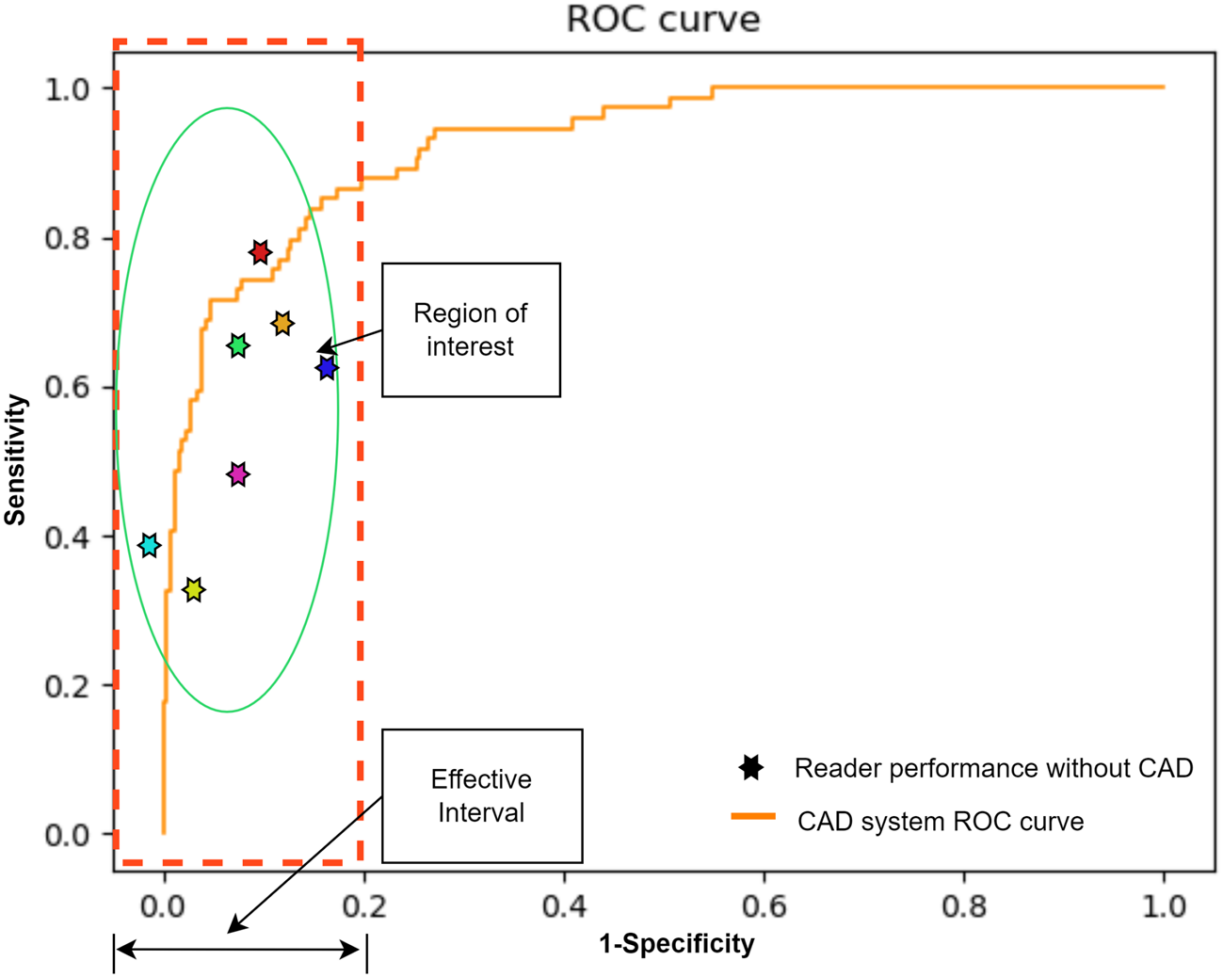
The effective interval delineates the region of practical significance within the ROC curve, specifically the area characterized by a False Positive Rate of less than 20% denoted as the effective interval. Enhancements beyond this region have negligible bearing on the practical performance of a commercial classification system. The region of interest denotes, the specific points on the curve where AUCReshaping is applied.

Figure 1 provides an overview of a typical abnormality detection scenario, as influenced by sensitivity and specificity. The figure illustrates multiple reader performances without assistance, along with that of a potential Computer-Aided Diagnostic (CAD) system at various classification thresholds (shaping the ROC curve). Given that one of the primary objectives of a CAD system is to reduce variation among readers in an assisted setting^14,15^, any enhancement in CAD system performance beyond the *effective interval* is unlikely to impact the reader’s performance in such a setting. The region of interest (ROI) highlights where performance improvement is of particular importance within the effective interval.

Frequently, practical systems are mandated to achieve an almost negligible false positive rate, primarily due to the substantial misclassification costs associated with the smaller abnormal or negative class. In medical applications, misclassifying an abnormal class, is akin to erroneously labeling a pathological image as normal, potentially leading to diagnostic delays and, in severe cases, endangering patient lives. For instance, we concentrate on a Chest X-Ray abnormality classifier designed to aid radiologists by effectively filtering out a maximal number of normal Chest X-Rays (CXRs), necessitating high sensitivity at high specificity. As depicted in Fig. 1, the classification thresholds within the Region of Interest (ROI) signify the number of misclassified positive samples while keeping the misclassifications of negative samples at a consistently low level (e.g., within 20%). This *effective interval* varies based on the specific application and system design. In the context of our CXR systems, we emphasize the 2–5% False Positive Rate (FPR) range as our region of interest.

While rank-based metrics have been proposed as a generalized solution to enhance sensitivity at high specificity^16^, there remains ample room for improving system performance. Past research has explored strategies like using an ensemble of classifiers and amplifying the cost of misclassifications for critical classes, aiming to maximize the overall AUC score and enhance the entire ROC curve. Similar research endeavors targeting the overall AUC score improvement, include approaches that address label noise by leveraging prior anatomical information^17^, such as heart-lung segmentation and other techniques to harness the high comorbidity of diseases in CXRs. Another approach looks into incorporating uncertainty scores^18^ alongside probabilistic estimates to enhance prediction robustness and accuracy. Recent research has also introduced matrix-instance-based one-pass AUC optimization^19^, mitigating the need to store the entire dataset or parts of it in memory while reducing run-time costs. The kernelized online imbalanced learning (KOIL) algorithm, a component of AUC maximization research^20^, proposes a non-linear classifier designed to maximize the AUC score for large imbalanced datasets. A novel approach to maximize AUC score, that is based on sampling mini-batches of positive/negative instance pairs^21^ and computing U-statistics to approximate a global risk minimization problem has also been shown to be simple, fast and learning-rate free. However, despite these advancements, these systems are not yet fully mature for deployment in the diagnostic industry^22^ and do not guarantee performance within the desired ROI.

In this paper, we,

- Propose a novel evaluation metric, known as sensitivity at high specificity, designed to assess the real-world performance of deep learning systems when dealing with data-imbalanced datasets.
- Introduce a mechanism to enhance the ROC-based performance metric through AUCReshaping, which is tailored for binary classification tasks.
- Evaluate the robustness of this method using both medical and non-medical datasets characterized by a significant class imbalance and skewed misclassification costs.

To elaborate, the AUCReshaping function amplifies the weights assigned to misclassified samples within the Region of Interest (ROI) on the ROC curve. In our application, this ROI corresponds to the high-specificity region. This enhancement is achieved through supervised fine-tuning, ensuring that the system maximizes the detection of positive samples, while minimizing the misclassification of negative class samples (False Positive Rate: FPR).

Furthermore, we initially explore the impact of the AUCReshaping function on a dataset of Chest X-Rays (CXRs). With the acquisition of millions of CXR images, the ability to confidently filter out normal images alleviates radiologists from the arduous task of parsing hundreds of images on a daily basis^14,15,23^. This, in turn, empowers them to direct their attention to critical patients, significantly expediting the diagnostic process, especially in situations akin to pandemics. These Self-Supervised Learning (SSL) based Computer-Aided Diagnosis (CAD) systems that support overburdened radiologists, not only enhance patient care but also contribute to reducing healthcare costs^24^-a particularly pressing concern today.

**Figure 2:**
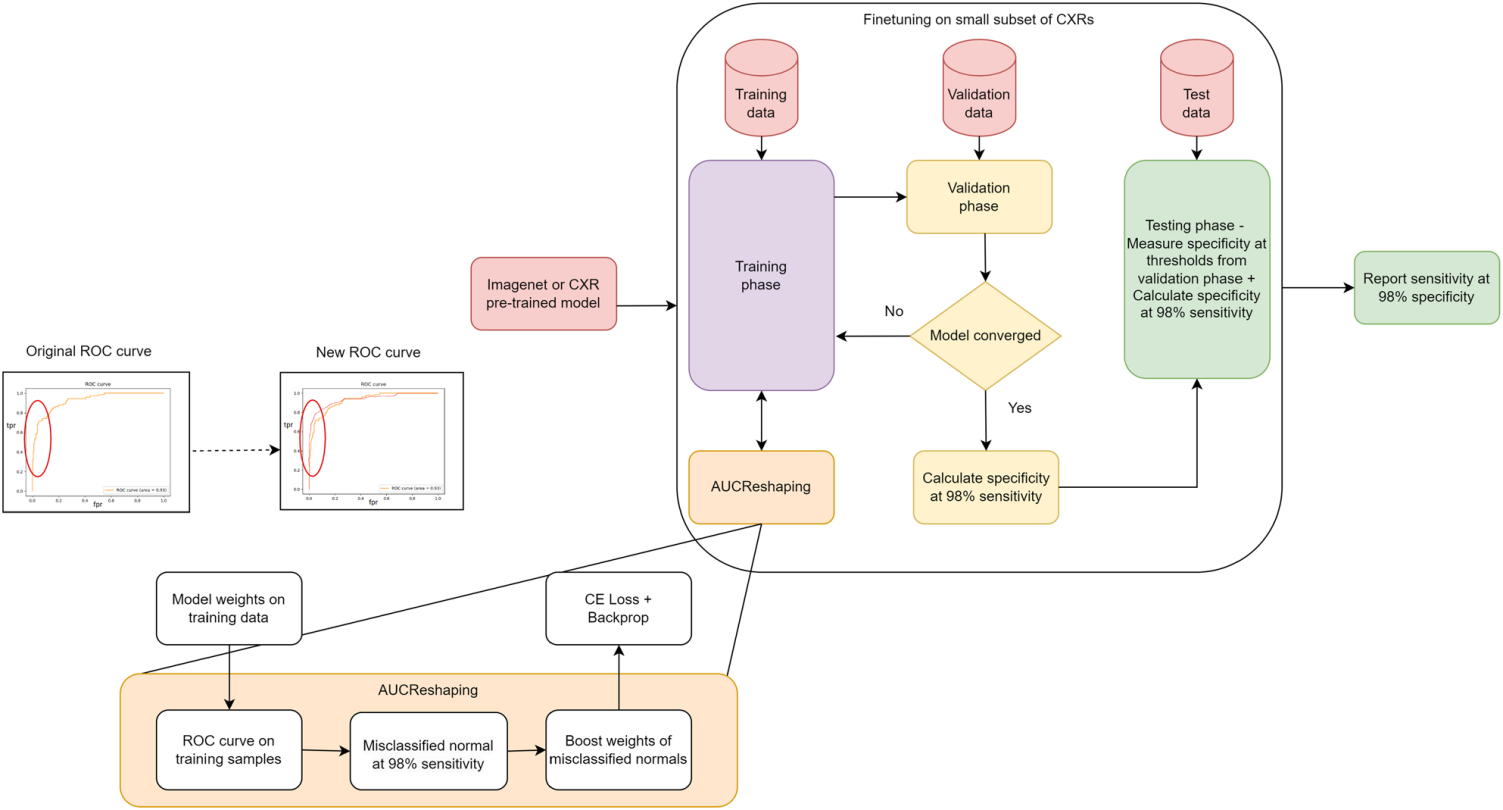
The schematic provides an overview of the experimental methodology, highlighting the AUC reshaping function’s role in reshaping a specific portion of the ROC curve, included in the “region of interest.” The ROC figures depicted in the diagram illustrate the adjustments made in the high-specificity region, with a slight potential decrease in the overall AUC value, resulting from modifications in the remaining parts of the curve.

In light of the recent availability of extensive CXR datasets such as NHS CXR-14^25^, CheXPert^26^, or MIMIC-II^27^, the accurate annotation of CXRs remains a significant challenge. In comparison to the large Deep Learning (DL) models, trained on billions of natural images sourced from social media with crowd-labeled annotations^28^, CXR annotations are considerably more time-consuming, expensive, and necessitate the expertise of skilled radiologists. As such, this paper investigates the application of AUCReshaping in conjunction with an SSL-based abnormality classifier, with the aim of developing a scalable and pragmatic solution for CXR CAD. Figure 2 provides an overview of the experiment’s general workflow, which involves the application of AUCReshaping in the fine-tuning stage on the training set. In this process, a pre-trained model undergoes fine-tuning using a subset of images from the pre-training dataset. During this phase, the AUCReshaping function identifies positive class samples that are misclassified at the high-specificity threshold, and amplifies their weights. The loss value is subsequently computed and backpropagated through the network. The high-specificity threshold determined during validation is carried over to the testing phase, where it serves as the actual classification threshold. The ROC curves on the left side of the Fig. 2 illustrate the modification of a potential high AUC curve to achieve the desired high sensitivity results.

## Methods

The paper describes scientific research using retrospectively acquired anonymized data. The anonymization was performed in accordance with applicable laws and regulations before secure transfer was made to Siemens Healthineers for the study. Use of the data followed all the applicable license terms. The study does not involve any clinical or human subject research component to it. As such, IRB approval and informed consent are not applicable because there are no data privacy issues and no patients were impacted by the research (did not affect treatment or diagnosis). The research was conducted conforming to the appropriate scientific practices and in accordance with the relevant guidelines and regulations of the institution conducting the experiments.

In this study, we introduce a novel function known as AUCReshaping, designed to re-weight the output predictions exclusively for misclassified training samples within the Region of Interest (ROI) of the ROC curve. As previously mentioned, in our specific application, this ROI corresponds to the high-specificity region (90–98%). High-specificity thresholds on the ROC curve are defined as the operating points that result in low False Positive Rates (FPR).

The AUCReshaping method is implemented during the fine-tuning stage, where it adapts the weights for the downstream task. This re-weighting process can be likened to boosting, where the weights of selected samples from the training set are iteratively increased. When dealing with two classes, we only boost the weights of samples with the lower misclassification cost, that are misclassified at the high-specificity threshold. The magnitude of boosting is determined based on a piece-wise approximation of the average distance of misclassified positive class samples from the decision boundary. The net effect is an increase in the uncertainty of the output predictions for misclassified positive samples, specifically the false negative samples. This adjustment is made while keeping the number of false positives at a low and fixed value, as demonstrated in Table 1. It is worth noting that this algorithm is not confined to a single threshold or boosting value. Instead, a modulating adaptive boosting value can be applied at each threshold, allowing for the specification of varying costs for these samples.

**Table 1:** Desired confusion matrix with True Positive(TP), True Negative (TN), False Positive (FP), False Negative (FN).

	Predicted 0	Predicted 1
Actual 0	TN	FP—fixed 2%
Actual 1	FN—reduce	TP

## AUC reshaping

The cost function corresponding to the proposed modification to the loss function is currently implemented only as a constant boosting value. This is mathematically described as

1 $$\begin{aligned} l = - \sum _i{(y_i\log (p_i-b_i) + (1-y_i)\log (1-p_i+b_i))}\hspace{5.0pt}, \end{aligned}$$

where

$$\begin{aligned} b_i= {\left\{ \begin{array}{ll} n, &{} \text {if }y_i==1\text { and }p_i\,<\,theta_{max} \\ 0, &{} \text {otherwise}. \end{array}\right. } \end{aligned}$$

*n* is the scalar boosting value and $theta_{max}$ is the high-specificity threshold such as the classification threshold at 0.95 or 0.98 specificity. Here, $y_i$ is the target label, $p_i$ is the output predicted from the network and $b_i$ is the boosting value applied conditionally as shown above. In this paper, we use $b_i$ as a constant value which is applied when the positive class is misclassified at the specified boosting thresholds on the ROC curve. The final loss function is an extension of the regular cross-entropy (CE) loss, incorporating an additional bias value for the selected samples. As mentioned earlier, the boosting can be applied at various thresholds on the ROC curve, which results in the adjustment of weights assigned to the misclassified positive samples based on their proximity to the high-specificity decision boundaries. Moreover, these boosting values don’t have to be uniform across all thresholds, allowing for diverse weightings of samples. This can be implemented as a discrete or continuous function.

**Figure 3:**
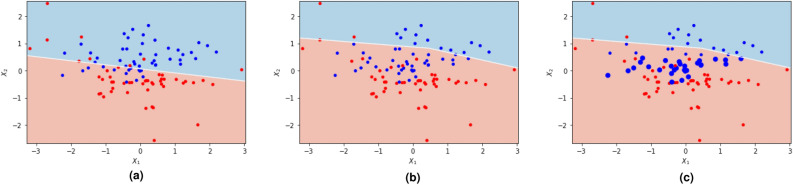
Illustration of the process of the AUCReshaping() function during fine-tuning. In each iteration, the function is applied to increase the weights of the misclassified samples at the high-specificity threshold. This process is repeated at multiple thresholds with different weighting values. (**a**) shows the optimal threshold that separates positive (blue) samples from negative (red) samples. (**b**) demonstrates a high-specificity threshold that aims to reduce the misclassifications of negative (red) samples. (**c**) represents the re-weighting of high-specificity misclassified positive samples (blue), which increases the uncertainty in the model’s predictions.

The AUCReshaping function can be better visualized in Fig. 3. In Fig. 3a, the diagram represents the typical optimal decision boundary that maximizes both sensitivity and specificity. Here, a few misclassifications of both the red and blue samples are observed, situated on the opposite side of the decision boundary. This choice of the classification boundary is conventional in a representative DL system and is commonly used to measure system performance by default. Figure 3b showcases the decision boundary at a specific high-specificity threshold. In this case, it has been adjusted to minimize misclassifications of the red samples. Although there is a higher number of misclassified blue samples, their associated cost is significantly lower than the opposite scenario. Hence, the decision boundary is shifted accordingly, resulting in a smaller percentage of misclassified red samples. Figure 3c highlights the misclassified positive samples that receive a boost from the AUCReshaping function, indicated by the larger blue circles. The network now focuses on these samples and learns relevant features to improve their correct classification, at this specific decision boundary. By incorporating different boosting values at various thresholds, the size of the boosted blue sample circles can vary, depending on the chosen thresholds. This flexibility enables the network to concentrate on particularly challenging samples. The choice of these design parameters depends on the specific application and data distribution. Although the current implementation does not parameterize the boosting value, it could also be approximated as a weighted moving average of the mean distance of misclassified samples to the decision boundary at high specificities.

In this study, we evaluate the performance of AUCReshaping using pre-trained models based on the Momentum Contrast Encoder (MoCo)^29^ and Swapping Assignments Between Views (SwAV)^30^, both of which are contrastive self-supervised learning methods. MoCo, an extension of the initial contrastive learning methodology, leverages a momentum encoder and additional data augmentations to achieve superior performance in various detection and segmentation tasks when compared to its Imagenet-supervised counterpart^29^. SwAV, on the other hand, is a self-supervised learning approach inspired by contrastive learning principles but differs in not using negative samples or pairwise comparisons between samples^30^. These publicly available pre-trained models, based on Imagenet^31^, (a dataset containing over 14 million natural images), serve as a basis for our evaluation. Additionally, we introduce a model pre-trained on 1.3 million Chest X-Rays (CXRs)^32^, which we also use for comparative purposes.

The classification task at hand is a binary classification problem, involving two categories of images. Specifically, it entails distinguishing between images related to a specific disease and other images. This task can be viewed as a detector for normal X-Rays, where X-Ray images associated with the disease are considered the negative class, and normal X-Ray images are the positive class that needs to be detected. To assess the impact of AUCReshaping, we evaluate the AUC score, sensitivity at both 95% and 98% specificity levels, on both the validation and test datasets across all our experiments.

In a practical application, following the training phase, we measure sensitivities at high-specificity levels on the validation dataset. The determined thresholds from the validation set are then used to assess sensitivity and specificity on the test set, as indicated in Fig. 2. This process yields a binary classification threshold, that achieves both high sensitivity and specificity on the test dataset. However, it’s important to note that the test set specificity levels may not perfectly align with the validation set levels, which can make it challenging to determine whether any improvements are solely due to the selection of a different point on the ROC curve. To address this, we independently compare the test set sensitivities at 95% and 98%, to evaluate whether the ROC curve has indeed improved. It’s worth emphasizing that achieving the right specificity level on the test data requires appropriate calibration of the validation thresholds.

**Figure 4:**
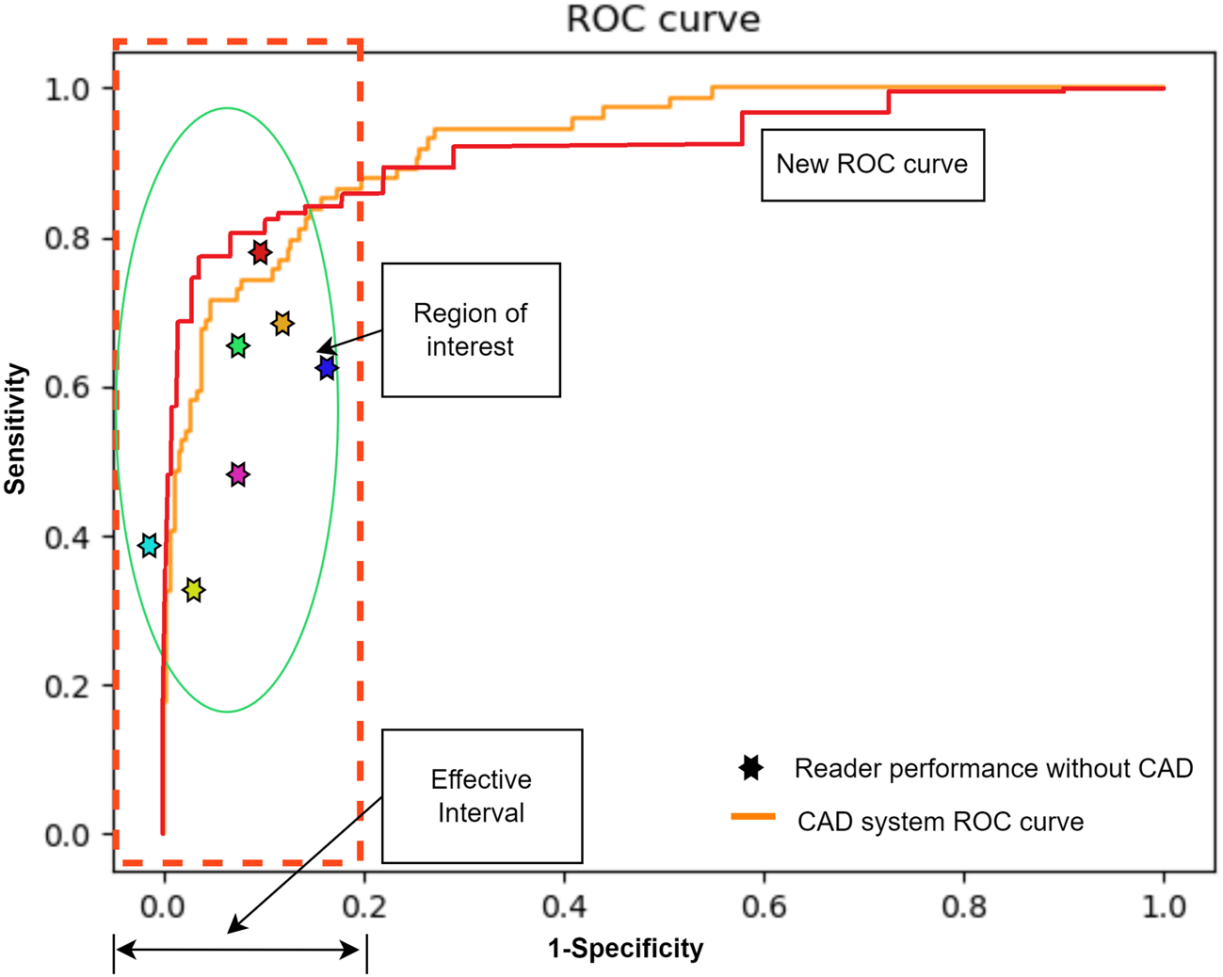
The original ROC curve of an SSL system (shown in orange) is transformed by AUCReshaping, resulting in the new ROC curve (depicted in red). While the final AUC score may experience a marginal reduction, it’s evident that this transformation leads to higher sensitivity at high-specificity levels.

The desired ROC curve is depicted in Fig. 4.We notice that the red curve may exhibit a slightly lower AUC score compared to the original orange curve. However, the performance in the high-specificity region is enhanced in the updated curve as indicated. Our proposed methodology offers a method for enhancing the operational characteristics of the deployed system by enabling improvements within a targeted segment of the ROC curve. To note, is that the ROC curve may be minimally negatively impacted in the region outside the effective interval.

### Data description

**Figure 5:**
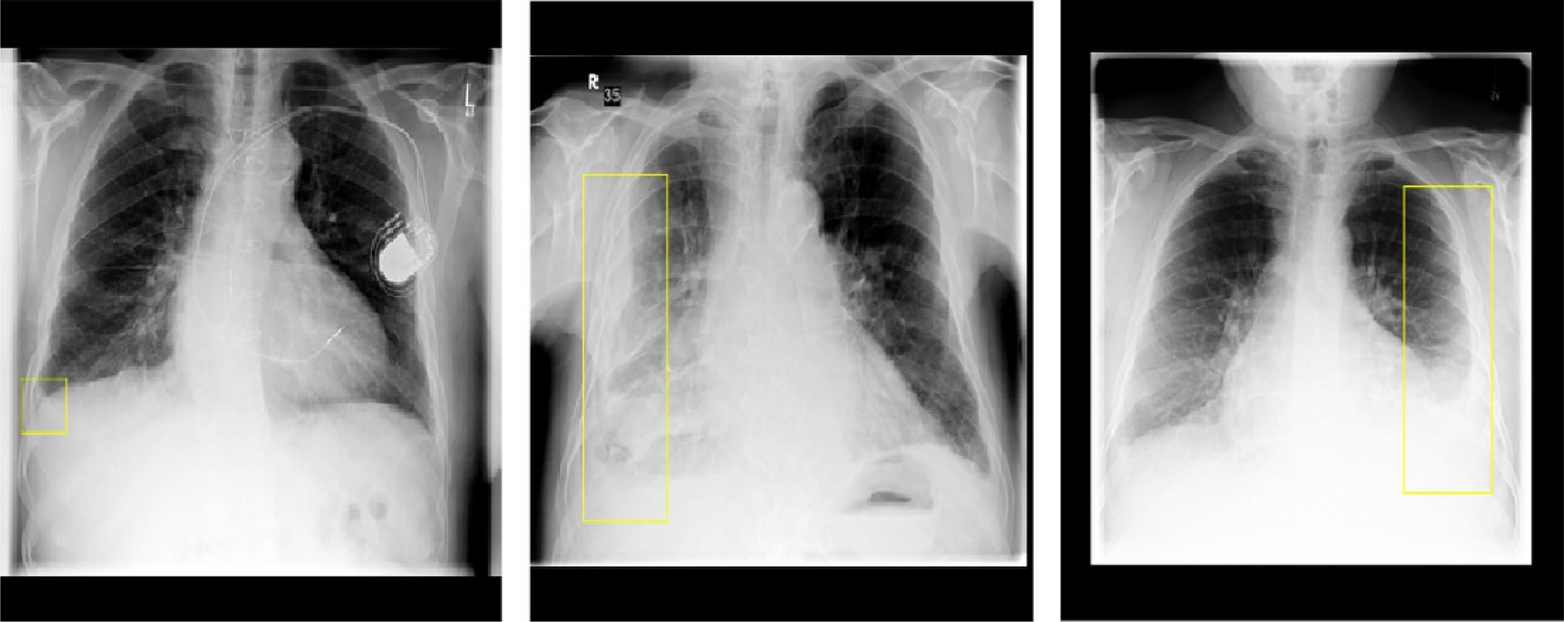
Example Chest X-Rays, sourced from the internal dataset CXR_16k, display pleural effusion abnormalities identified by bounding boxes.

The CXR dataset used in the fine-tuning stage comprises 16,953 anonymized training images and a few example images are shown in Fig. 5. The images indicate typical anonymized frontal view CXR images of patients with bounding boxes indicating the presence of pleural effusion. A subset of this dataset is used as a validation dataset, where the images are not part of the training set. The CXR dataset includes 3,663 pleural effusion, 1772 pneumothorax (ptx) images, and 14 other abnormalities. The images are stored in 10-bit DICOM format and vary in resolution, typically exceeding 2048x3096 pixels. An expert panel of radiologists provided bounding box coordinates, each associated with a disease label, and a single image could have multiple annotated boxed regions belonging to different categories.

To create image-level labels indicating the absence or presence of abnormalities, a binary coding scheme was employed (1=absent, 0=present). The training dataset remains consistent across all experiments, but the ground truth labels differ according to the specific classification task. For instance, in the first case a pleural effusion list is formed with images containing pleural effusion marked as 0 and images without marked as 1. A similar procedure is followed to create the ptx list and in the third case, a combined training list is formed, where an image with either ptx or pleural effusion is labeled as class 0, while an image without either of them is assigned class 1. This results in 3223, 1607, and 4069 positive training samples for the pleural effusion, ptx and combined classification task, respectively. The validation dataset exhibits a similar pattern, containing 440, 165, and 466 positive samples, as indicated in Table 2. There could be multiple images of a patient in the training dataset.

A similar procedure is applied to generate the test datasets. The pleural effusion test dataset comprises 331 images, including 74 pleural effusion images and 257 images featuring other abnormalities. In the case of pneumothorax (ptx), a test dataset with 412 images is established, containing 154 ptx images and 258 non-ptx images. Additionally, a combined test dataset is created, incorporating 228 images that exhibit either “pleural effusion or ptx” and 294 images without either of these diseases, resulting in a total of 522 test images. The validation dataset is a subset of the training dataset, where the class-wise ratio is maintained. Particular care is taken to ensure that no images from the test set patients are included in either the training or validation set. The complete data split is depicted in Table 2.

The AUCReshaping methodology is further evaluated using the VinDr-Mammo dataset, an extensive benchmark dataset designed for full-field digital mammography^33,34^. This dataset encompasses a total of 5000 full-view examinations, including craniocaudal (CC) and mediolateral oblique (MLO) views, thus comprising a total of 20,000 images. Notably, the dataset features consensus annotations provided by three experienced radiologists, particularly for assessments based on the Breast Imaging Reporting and Data System (BI-RADS) and breast density categorization^35^.

Within the VinDr-Mammo dataset, a training set of 16,000 images is available. This training set is divided into two subsets: a training subset comprising 13,600 images and a validation subset containing 2400 images. The test set consists of the original 4,000 images from the dataset. These images are acquired from various mammography systems, including those from manufacturers such as Siemens, Planmed, and IMS. The selection of images for both the training and validation subsets is made at the patient level, with meticulous attention to ensuring an even distribution of samples based on their class categories (BI-RADS and breast density). This careful curation aims to mitigate potential sources of bias in the experiments.

Our study leverages this dataset to explore two distinct classification tasks: breast density categorization and BI-RADS classification. In the context of breast density categorization, the four breast density categories, ranging from A (almost entirely fatty) to D (extremely dense)^35^, are further sub-categorized into two classes. The positive class encompasses non-dense breasts (categories A and B), while the negative class comprises dense breasts (categories C and D)^36,37^. In the training, validation, and test splits, we have 1380, 228, and 400 samples for the positive class, and 12,220, 2172, and 3600 samples for the negative class, respectively. Additionally, the BI-RADS assessment scores are categorized into two classes based on the BI-RADS management recommendations for tissue diagnosis^35^. The positive class is composed of images with BI-RADS scores of 4 and 5, while the negative class includes images with BI-RADS scores of 1, 2, and 3. Across the training, validation, and test sets, we have 656, 134, and 198 images in the positive class, and 12,944, 2266, and 3802 images in the negative class, respectively. Notably, both tasks involve the positive classes serving as the minority abnormality class, entailing a high misclassification cost. Furthermore, it is observed that data imbalance is more pronounced in the BI-RADS task compared to the breast density task.

**Figure 6:**
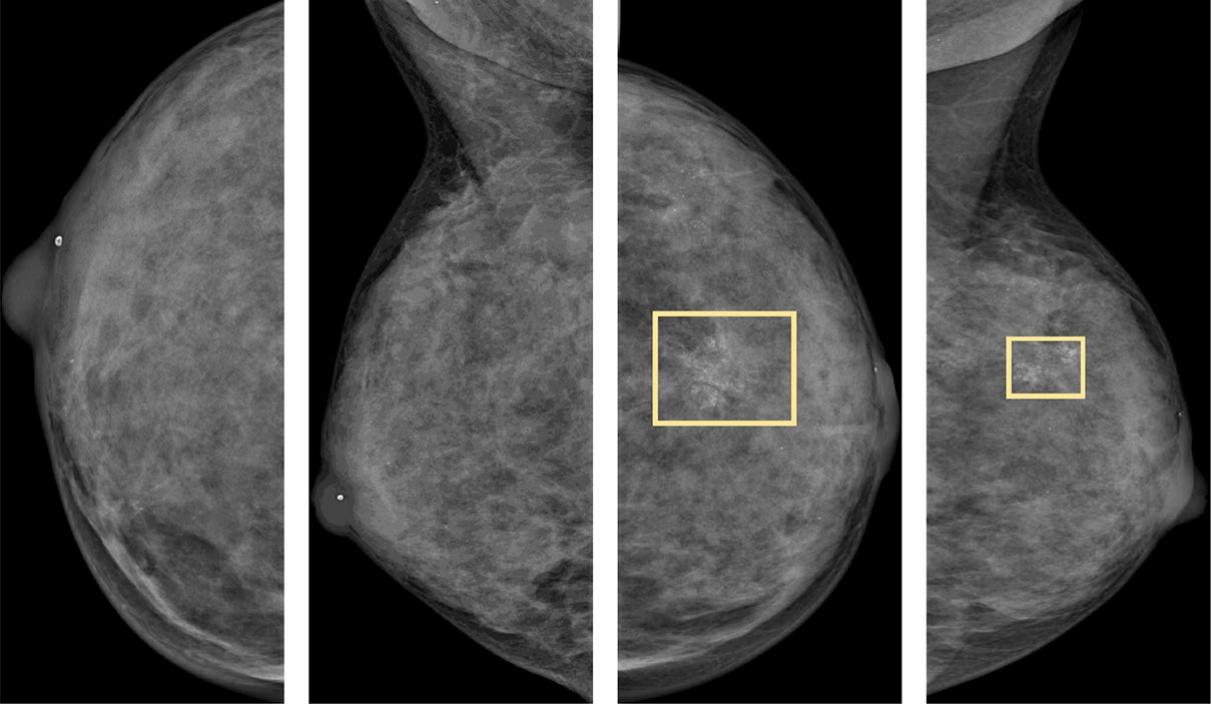
Breast mammogram images featuring bounding boxes highlighting calcification and a mass abnormality, as sourced from the VinDr-Mammo dataset.

For illustrative purposes, Fig. 6 showcases breast mammogram images from a 42-year-old patient within the VinDr-Mammo dataset^33^. These images display the R-CC, R-MLO, L-CC, and L-MLO views, presented from left to right. Both breasts exhibit a breast density category of D, with the right breast (R) assigned a BI-RADS score of 2, and the left breast (L) designated with a BI-RADS score of 5. These ratings are attributed to the presence of suspicious calcifications and a mass, as indicated within the bounding box.

To further validate the robustness of this method in non-medical domains, we applied AUCReshaping to the domain of credit card fraud detection. The dataset used comprises transactions made by European cardholders and spans over a two-day period^38–45^. Within this dataset, 492 transactions are fraudulent, while 284,807 are considered normal transactions, accounting for only 0.172% of all transactions. The dataset was divided into training and test sets, with 227,454 and 56,861 positive samples in each, and 391 and 101 negative samples, respectively. The total number of images in the datasets along with the respective number of positive samples (enclosed in brackets) are visually represented in Table 2.

**Table 2:** List of datasets used for classification studies in this paper.

Name	Training	Validation	Test
	Total (positive)	Total (positive)	Total (positive)
CXR_16k_DICOM (pleural effusion)	15,453 (3223)	1500 (440)	331 (74)
CXR_16k_DICOM (ptx)	15,453 (1607)	1500 (165)	412 (154)
CXR_16k_DICOM (ptx + pleural effusion)	15,453 (4069)	1500 (466)	522 (228)
VinDr-Mammo Breast Density	13,600 (1380)	2400 (228)	4000 (400)
VinDr-Mammo BI-RADS	13,600 (656)	2400 (134)	4000 (198)
CreditCardFraudDetection	227885 (391)	–	56962 (101)

### Implementation details

In the CXR experiments, images are first resized to 256x256 pixels before being input into the fine-tuning stage. These experiments are executed on 8 GPUs with a batch size of 64, employing PyTorch and Distributed Data-Parallel computing (DDP). The models are optimized using the Adam^46^ optimizer, initialized with a learning rate of 0.01, weight decay set at 1e-5, and a Cross-Entropy (CE) loss function. Class weights are determined based on the class ratio within the training dataset^30^. Distributed weighted sampling is adopted to over-sample the negative classes to address class imbalance and ensure smooth data loading across the GPUs.

The number of repeated measurements follow a negative binomial distribution. The number of repeated measurements needed for a high-enough statistical power (>0.98) is calculated using the technique described in^47^ known as “marginal benefit”. Based on the statistical power, top 5 models were averaged for all experiments. The baseline experiments were repeated 8-10 times, and the rest are repeated only five times to calculate the average. In the end-to-end (e2e) fine-tuning process, the training loss gradually diminishes until overfitting occurs. Consequently, the epoch with the lowest validation loss, often occurring within 10 epochs, is selected. As mentioned earlier, a range of pre-trained models are assessed.

Augmentations are typically applied only in the training stage and not in the validation or testing stage. Working with DICOM images necessitates the application of a distinct set of augmentations. Given the higher precision of the input data, the built-in PyTorch transforms are not directly applicable and must be re-implemented to suit the data format. Augmentations like random intensity scaling and horizontal flipping are utilized, in addition to a custom normalization process for the DICOM images. This normalization includes histogram equalization and dynamic window scaling^32^.

For the breast density experiments, the images are resized to 256 $\times$ 256 pixels before they are input into the fine-tuning network. In the case of DICOM images, pre-processing is carried out initially to remove the CC and MLO view labels. These images contain labels indicating the right or left breast, along with the corresponding CC or MLO view superimposed on the image. These labels are first cropped, and fundamental array operations are employed to remove the black background. Subsequently, the pixel values are re-scaled using the window level and width from the DICOM data, resulting in images saved in PNG format with standard aspect ratios and sizes. Unlike CXRs, mammogram images employ different augmentations, specifically random equalization and random erasing, with low probabilities. Other network hyper-parameters align with those used in the CXR experiments, and the results represent the average of 5 runs. Evaluation of the classification results is performed using a SwAV-based Imagenet pre-trained model.

For the credit card fraud classification, a straightforward Multi-layer Perceptron (MLP) Network is employed, comprising a combination of linear, ReLU, and dropout layers with a dropout probability of 0.5. Experiments are executed on a single GPU, with a batch size of 100, and a simple SGD optimizer is utilized. To address the data imbalance, positive_weights of 5 are applied in conjunction with the BCEWithLogitsLoss function. The model undergoes training for 200 epochs until convergence of the training loss, and the results represent the average of 5 independent runs.

## Results

The models used to test AUCReshaping on CXRs are called “SSL Imagenet SwAV pre-trained”, “SSL Imagenet MoCo pre-trained” and “SSL CXR_1million SwAV pre-trained” respectively in the Tables 3, 4 and 5. All of the experiments are conducted with ResNet-50 architecture base models. The CXR experiments are conducted for two different abnormality classifications, specifically targeting pleural effusion and pneumothorax. These classifications include pleural effusion versus non-pleural effusion, ptx versus non-ptx, and a combined dataset for normal versus abnormal classification, where “normal” denotes images without either pleural effusion or ptx. Pleural effusion is relatively easy for the classifier to detect, resulting in higher baseline AUC scores, while ptx poses a more challenging detection task^48^. These two categories represent opposite ends of the difficulty spectrum and collectively provide a comprehensive assessment of the system’s performance in various abnormality detection scenarios.

### Pleural effusion

Table 3 displays the outcomes of employing the AUCReshaping function with various boosting values applied at specific thresholds. The sections in the table represent results associated with different pre-trained models, while the rows represent outcomes from different experiments. In the first column, we present the boosting values applied, and in the second column, the corresponding thresholds from the ROC curve during training. For e. g., the configuration [0.4, 0.2, 0.1, 0.1] at [0.90, 0.92, 0.95, 0.98] signifies that a boosting value of 0.4 is applied at the 0.90 threshold, 0.2 at the 0.92 threshold, and 0.1 at both the 0.95 and 0.98 thresholds. These boosting values are employed to modify the loss values or output logits at multiple boosting thresholds. The sensitivity scores with different pre-trained models are compared, with the highlighted rows indicating the specific boosting values and thresholds that lead to the highest improvements. The asterisk indicates results with statistically significant improvements at *p*< 0.05. Colored values represent the percentage change when comparing a given row to its corresponding baseline. The skewness and kurtosis for all the measured sensitivity values is reported in brackets. This format is maintained for all the experiments henceforth. Notably, the choice of employing a constant boosting value or a set of increasing or decreasing values yields distinct results.

**Table 3:** Pleural effusion versus Non-pleural effusion sensitivity at high-specificity values on CXR_16k before and after reshaping the ROC curve when all layers are retrained (e2e fine-tuning).

	Boosting values	Boosting thresholds	AUC score	Sensitivity @0.95 Specificity	Sensitivity @0.98 Specificity
				(Skewness, Kurtosis)	(Skewness, Kurtosis)
SSL Imagenet SwAV pre-trained					
Baseline 1	0		0.979±0.005	0.902±0.02	0.802±0.11
				(0.00296, − 2.56558)	(0.10704 , − 2.39901)
AUCReshaping	1	0.95	0.979±0.003	0.908±0.02	0.867±0.03*
				( 0.00325, − 2.56501)	(0.01025, − 2.55059)
AUCReshaping	1	0.90,0.95,0.98	0.98±0.002	0.909±0.04	0.867±0.05*
				(0.01164, − 2.42355)	(0.02602, − 2.39705)
AUCReshaping	0.2	0.90,0.92,0.95,0.98	0.98±0.002	0.904±0.03*	0.839±0.04
				(0.00589, − 2.55968)	(0.02010, − 2.53102)
% change			*0.1%*	*0.20%*	*4.67%*
AUCReshaping	[0.1,.0.1,0.2,0.4]	0.90,0.92,0.95,0.98	0.98±0.002	0.921±0.01*	0.809±0.05
				(0.00175, − 2.56794)	(0.02950, − 2.51374)
AUCReshaping	**[0.4,0.2,0.1,0.1]**	**0.90,0.92,0.95,0.98**	0.982±0.002	**0.926**±0.02*	**0.844**±0.03*
				(0.00332, − 2.56496)	(0.00972, − 2.55334)
% change			*0.3%*	*2.68%*	*5.24%*
SSL CXR_1million SwAV pre-trained					
Baseline 2	0		0.979±0.004	0.896±0.04	0.742±0.17
				(0.01158, − 2.54928)	(0.2659, − 2.21879)
AUCReshaping	0.2	0.90,0.92,0.95,0.98	0.979±0.003	0.907±0.03	0.811±0.03*
				(0.00609, − 2.55984)	(0.01109, − 2.54954)
% change			*0.04%*	*1.21%*	*9.24%*

The % change is from preceding row compared to corresponding baseline data. The asterisk (*) signifies statistical significance (*p*< 0.05) compared to the baseline.

The bolded rows are the results indicating best or highest performance. The italics is the positive % change of the previous row when compared to baseline.

Based on the experiments, it has been observed that the overall AUC score remains relatively stable, with the possibility of a slight decrease, (up to 5%), that depends on the extent of correction applied. Notably, a significant drop in the AUC score can have a detrimental impact on high-specificity sensitivities. It’s interesting to note that fine-tuning only the frozen ResNet-50 layers, leads to lower baseline AUC scores, but can result in higher improvements (from the AUCReshaping function), when compared to their corresponding end-to-end (e2e) fine-tuning experiments. Moreover, the percentage of improvement, at either discrete observation point can vary based on the specific boosting thresholds on the ROC curve.

The experiments aimed to identify the most effective method for boosting misclassified instances, whether through a constant value or an average of the output predictions. It was observed that using the average of the output predictions from the misclassified samples did not yield meaningful results. A more logical approach is to base the boosting criteria, on the distance of the average of the misclassified positive samples to the high-specificity decision boundary. Implementing a weighted moving average of this distance, appears to be the most appropriate metric for determining the boosting value. While a comprehensive grid search of all possible values was not conducted, preliminary experiments suggest that there is a “sweet spot” for the boosting value. Excessive boosting can lead to a drop in AUC and severe degradation in sensitivities.

In this context, Table 3 provides valuable insights, demonstrating a notable improvement of approximately 5% in sensitivity at 98% specificity when retraining all layers of the Imagenet SwAV pre-trained model. The improvements are even more substantial in the CXR_1million model, but this can be attributed to the baseline results not being close to saturation, leaving more room for improvement.

### Pneumothorax

The experiments were also replicated for the ptx versus non-ptx classification, where ptx was considered the negative class, and non-ptx was regarded as the positive class. This classification problem presents a greater challenge for the model due to the smaller regions of ptx that could be missed, when resizing the images to 256 × 256. This complexity is reflected in the lower baseline AUC values, with AUC at 0.93 compared to the 0.98 AUC for pleural effusion, as evident in Table 4.

**Table 4:** Pneumothorax versus Non-pneumothorax Sensitivity at high-specificity values on CXR_16k before and after reshaping the ROC curve, fine-tuned e2e.

	Boosting values	Boosting threshold	AUC score	Sensitivity @0.95 Specificity	Sensitivity @0.98 Specificity
				(Skewness, Kurtosis)	(Skewness, Kurtosis)
SSL Imagenet SwAV pre-trained					
Baseline 1	0		0.93±0.007	0.754±0.03	0.652±0.05
				(0.48277, − 2.23206)	(0.0403, − 2.49952)
AUCReshaping	[0.4,.0.2,0.1,0.1]	0.90,0.92,0.95,0.98	0.936±0.004	0.773±0.04	0.687±0.04
				(0.01656, − 2.53861)	(0.02293, − 2.52290)
AUCReshaping	**0.2**		**0.933**±**0.002**	**0.787**±0.03*	**0.769**±0.09*
				(0.38437, − 2.24314)	(1.15872, − 0.50352)
% change			*0.28%*	*4.30%*	*16.67%*
SSL CXR_1million SwAV pre-trained					
Baseline 2	0		0.913±0.008	0.643±0.16	0.666±0.16
				(0.00267, − 2.56611)	(0.0633, − 2.44322)
AUCReshaping	0.2	0.90,0.92,0.95,0.98	0.92±0.009	0.725±0.02*	0.631±0.06
				(0.00864, − 2.55419)	(0.55336, − 2.00594)
% change			*0.71%*	*12.69%*	***− 5.23%***

The % change is from the preceding row compared to corresponding baseline data. An asterisk (*) signifies statistical significance improvement over the baseline (*p*< 0.05).

The bolded rows are the results indicating best or highest performance. The italics is the positive % change of the previous row when compared to baseline and the bold italics is the negative % change of the previous row when compared to baseline.

The results for ptx classification presented in Table 4 demonstrate a 16% improvement in sensitivity at 98% specificity when using an Imagenet SwAV pre-trained model. Additionally, the highlighted results are statistically significant with *p*< 0.05. In contrast, the results obtained with the CXR_1million pre-trained model show a 5% decrease at the same point. This variation can be attributed to the choice of optimizing the 95% specificity as the operating point which indicate a statistically significant 12.69% increase with *p*< 0.5.

The experiments indicate that different boosting values can lead to varying levels of improvement, and determining the optimal boosting strategy falls outside the scope of this paper and is subject to system design considerations. As observed in previous experiments, the best results have been consistently achieved with the Imagenet pre-trained SwAV models.

### Pleural effusion + pneumothorax

The final set of CXR experiments involved a combined dataset, where the negative class consisted of all images with either pleural effusion or pneumothorax, and the positive class contained images with any other abnormality disease. The results are summarized in Table 5. Notably, improvements ranging from 2 to 12% were observed across various pre-trained models when retraining all layers. Additionally, the results using MoCo^49^ based Imagenet pre-trained models were compared, and the corresponding AUC scores were documented in Table 5. Of note, the MoCo-based Imagenet pre-trained model exhibited the highest AUC scores, when compared to the SwAV-based Imagenet pre-trained model and the SwAV-based CXR_1million pre-trained model. Here, the AUC score exhibited a marginal decrease of 0.23% when fine-tuning end-to-end, but the sensitivity showed improvements ranging from 3 to 18% at various points on the ROC curve. The asterisk indicates the statistically significant results with *p*< 0.05, and we see the MoCo based models, demonstrate the maximum improvement. The behavior of the AUC value aligns with expectations, given that the modified algorithm does not explicitly optimize the overall AUC in the loss function.

**Table 5:** 2-disease normals versus abnormal with sensitivities at high specificities on CXR_16k when fine-tuning e2e.

	Boosting values	AUC score	Sensitivity @0.95 Specificity	Sensitivity @0.98 Specificity
			(Skewness, Kurtosis)	(Skewness, Kurtosis)
SSL Imagenet SwAV pre-trained				
Baseline 1	0	0.904±0.003	0.696±0.04	0.462±0.03
			(0.02506, − 2.39922)	(0.04075, − 2.48752)
AUCReshaping	0.2	0.906±0.002	0.751±0.03*	0.492±0.05
			(0.00875, − 2.55406)	(0.07745, − 2.39887)
% change		*0.18%*	*7.98%*	*6.54%*
AUCReshaping	**0.15**	**0.904**±**0.002**	**0.684**±**0.03**	**0.518**±0.04*
			(0.01746, − 2.53772)	(0.50586,− 2.16919)
% change		*0.04%*	***− 1.71%***	*12.17%*
AUCReshaping	0.4, 0.2	0.90±0.003	0.711±0.01*	0.471±0.03
			(0.00185, − 2.56771)	(1.00735, − 1.21391)
% change		*0.11%*	*2.29%*	*1.95%*
AUCReshaping	0.8,0.4	0.908±0.003	0.701±0.03	0.506±0.05
			(0.01353, − 2.54623)	(1.10514, − 0.83250)
% change		*0.42%*	*0.85%*	*9.57%*
SSL CXR_1million SwAV pre-trained				
Baseline 2	0	0.905±0.002	0.692±0.04	0.433±0.04
			(0.48913, − 2.22105)	(0.49732, − 2.23369)
AUCReshaping	**0.15**	**0.906**±**0.005**	**0.710**±**0.03**	**0.485**±0.02*
			(0.48147, − 2.24463)	(0.01108, − 2.54916)
% change		*0.09%*	*2.66%*	*12.11%*
SSL Imagenet MoCo pre-trained				
Baseline 3	0	0.914±0.005	0.726±0.04	0.485±0.04
			(0.01966, − 2.32952)	(0.59124, − 1.88763)
AUCReshaping	**0.4,0.2**	**0.912**±**0.005**	**0.755**±0.01*	**0.575**±0.08*
			(0.00293, − 2.56572)	(0.1315 ,− 2.34931)
% Change		***− 0.23%***	*3.88%*	*18.52%*

The % change is from the preceding row compared to the corresponding baseline data. The asterisk (*) signifies statistical significance (*p*< 0.05) compared to the baseline.

The bolded rows are the results indicating best or highest performance. The italics is the positive % change of the previous row when compared to baseline and the bold italics is the negative % change of the previous row when compared to baseline.

### Mammogram classification

The results presented in Table 6 pertain to two distinct classification tasks: breast density and BI-RADS category classification, both employing a SwAV-based Imagenet pre-trained model. For breast density classification, the baseline AUC score is notably high, registering at 0.97. Consequently, the sensitivity at 95% and 98% specificity is already above 0.8, resulting in only a marginal increase in sensitivity at both thresholds.

In contrast, the AUC score for the BI-RADS classification task is considerably lower, resting at 0.77. This leads to lower sensitivities at high-specificity levels, namely 0.18 and 0.08. Significant improvements are observed in this scenario, with a 19.64% increase in sensitivity at 95% specificity, and an impressive 42.33% increase at 98% specificity. These improvements elevate sensitivity from 0.18 to 0.2 at 95% specificity and from 0.058 to 0.09 at 98% specificity. Again, the asterisk indicates the statistical significance of improvement over the baseline with *p*< 0.05.

### Credit card fraud classification

Similarly, the results in Table 7 underscore the effectiveness of AUCReshaping in the context of credit card fraud detection. Here, the baseline AUC score of 0.98 is considered excellent and approaches the current gold standard. Despite this, it still translates to a notable number of false positive misclassifications—normal transactions falsely labeled as fraudulent—when striving to maximize fraudulent classifications, i.e., TNR. We examine the sensitivities at much higher thresholds than before, considering nearly 0 fraudulent transactions are allowed to be misclassified. At the 99% and 99.99% specificity thresholds, the baseline sensitivities are measured at 0.73 and 0.33, respectively. However, after applying AUCReshaping, significant improvements are observed, with a 3.05% increase in sensitivity at 99% specificity and an encouraging 27.4% increase at 99.99% specificity. This translates to almost 10,000 fewer misclassifications of normal credit card transactions, as fraudulent transactions at the 99.99% specificity threshold. These results underscore the potential of AUCReshaping in reducing false positives and improving the sensitivity of fraud detection systems. In Table 7 the asterisk denotes a a statistically significant improvement over the baseline with *p*< 0.1

**Table 6:** Breast mammogram classification using Imagenet pre-trained SwAV model with e2e fine-tuning.

	Boosting values	Boosting threshold	AUC score	Sensitivity @0.95 Specificity	Sensitivity @0.98 Specificity
				(Skewness, Kurtosis)	(Skewness, Kurtosis)
Breast density classification A, B versus C, D					
Baseline 1	0		0.969±0.001	0.882±0.006	0.810±0.017
				(0.27195, − 2.21143)	(0.00308, − 2.30251)
AUCReshaping	0.1	0.90,0.92,0.95,0.98	0.969±0.001	0.885±0.006	0.804±0.012
				(0.00028, − 2.36314)	(0.00143, − 2.36109)
AUCReshaping	**0.1**	**0.90,0.92,0.95,0.98**	**0.969**±**0.012**	**0.883**±**0.03**	**0.824**±0.09*
				(0.00033, − 2.26612)	(0.00124, − 2.30559)
% Change			*0%*	*0.04%*	*1.8%*
BI-RADS classification task Class 1,2,3 versus Class 4, 5					
Baseline 2	0		0.77±0.007	0.18±0.03	0.058±0.03
				(0.43988, − 1.65774)	(1.09271, 0.49327)
AUCReshaping	0.2	0.90,0.92,0.95,0.98	0.76±0.01	0.15±0.02	0.07±0.02
AUCReshaping	**0.1**	**0.95**	**0.775**±**0.01**	**0.2**±**0.04**	**0.09**±0.02*
				(0.59608, − 1.36873)	(0.40236, − 1.46025)
% Change			*1.1%*	*19.64%*	*42.35%*

Classification performed on two tasks, namely, breast density binary classification (Density A, B vs. Density C, D) and BI-RADS binary classification (BI-RADS 1, 2, 3 vs. BI-RADS 4, 5). The asterisk (*) signifies statistical significance (*p*< 0.05) compared to the baseline.

The bolded rows are the results indicating best or highest performance. The italics is the positive % change of the previous row when compared to baseline.

**Table 7:** Credit card fraud classification of normal versus fraudulent transactions.

	Boosting values	Boosting threshold	AUC score	Sensitivity @0.98 Specificity	Sensitivity @0.9999 Specificity
				(Skewness, Kurtosis)	(Skewness, Kurtosis)
Baseline 1	0		0.98±0.0004	0.73±0.007	0.33±0.005
				(0.00058, − 2.57028)	(1.00937, − 1.23028)
**AUCReshaping**	**0.2**	0.90,0.92,0.95,0.98,0.99	**0.98**±**0.0012**	**0.76**±**0.03**	**0.42**±0.07*
				(0.01040, − 2.55159)	(0.20175, − 2.19042)
% change			*0.17%*	*3.05%*	*27.37%*

The asterisk (*) signifies statistical significance (*p*< 0.10) compared to the baseline.

The bolded rows are the results indicating best or highest performance. The italics is the positive % change of the previous row when compared to baseline.

### Extension to multi-class and multi-label

We extend the application of our metric to the multi-class scenario, wherein each sample is associated with a single class, and the sum of predicted probabilities always equals 1. Our investigation focuses on the effect of AUCReshaping within the context of the BIRADS classification task. Specifically, we reshape the ROC curve for the BIRADS-5 category, representing the most severe malignant class. Notably, we observe a substantial enhancement of 35% in sensitivity at a specificity of 98%. It’s important to note that our study primarily addresses AUC as a one-versus-all metric for BIRADS-5, and the macro average AUC and accuracy may be negatively impacted. To refine our method, we introduce modifications aimed at augmenting the weights of misclassified samples and diminishing the weights of the highest probability within the remaining four categories, ensuring that the total probabilities sum to 1. The results as denoted in Table 8, show a 0.66% improvement in the AUC score for BIRADS-5 as well as a 36.42% improvement in the sensitivity at 98% specificity. The results demonstrate the potential of AUCReshaping in a multi-class scenario as well. For future work, we propose to showcase these enhancements and anticipate conducting a more extensive and meticulous investigation encompassing the combination of multiple classes (such as reducing the misclassification rate of both BIRADS-4 and BIRADS-5 together) and the assessment of overall classification accuracy, macro average AUC and F1 scores.

In the multi-label scenario, samples may be associated with multiple labels, exemplified by cases involving the presence of both pleural effusion and pneumothorax within the same image. In such instances, the treatment of ROC curves per label independently, aligns with the binary classification methodology employed in our study.

**Table 8:** Multi-class classification results of BIRADS-5 category in breast mammogram classification.

	Boosting values	Boosting threshold	AUC score	Sensitivity @0.95 Specificity	Sensitivity @0.98 Specificity
				(Skewness, Kurtosis)	(Skewness, Kurtosis)
Baseline 1	0		0.9726±0.004	0.9006±0.03	0.5064±0.12
				(1.03042, − 1.18808 )	(1.31477, 0.09810)
AUCReshaping % change	**0.2**	**0.90,0.92,0.95,0.98**	**0.979**±0.002*	**0.87**±**0.04**	**0.69**±0.09*
				(0.01627, − 2.41271)	(0.11340, − 2.24435)
			*0.66%*	***− 3.42%***	*36.42%*

The ROC curve of only category 5 as the critical class is reshaped. The asterisk (*) signifies statistical significance (*p*< 0.05) compared to the baseline.

The bolded rows are the results indicating best or highest performance. The italics is the positive % change of the previous row when compared to baseline and the bold italics is the negative % change of the previous row when compared to baseline.

## Discussion and conclusion

The evaluation of deep learning systems on heavily imbalanced datasets often requires practical metrics beyond the traditional AUC score to gain a more comprehensive understanding of system performance. In response to this need, we introduce a novel metric known as “sensitivity at high-specificity” This metric is designed to assess the performance of deployed systems and enables the optimization of system performance at specific operating points along the ROC curve.

To further enhance system performance, we present a new algorithm called AUCReshaping. This algorithm is applied iteratively during the fine-tuning phase when adapting pre-trained models to domain-specific classification tasks. AUCReshaping aims to improve sensitivity at high-specificity thresholds, addressing the challenges posed by imbalanced datasets and providing a more practical and effective approach to evaluating and optimizing deep learning systems.

In this paper, we explore the effectiveness of AUCReshaping on three distinct datasets: Chest X-Rays (CXRs), breast mammograms, and credit card fraud detection. Our CXR experiments encompass three classification tasks: pleural effusion versus non-pleural effusion, pneumothorax (ptx) versus non-ptx, and a combined classification for images containing either pleural effusion or ptx as a negative class. To perform these tasks, we leverage a range of pre-trained models, including Imagenet-based models such as SwAV and MoCo, as well as an internally pre-trained model, referred to as CXR_1million. The general workflow includes a pre-training and fine-tuning stage, where fine-tuning is carried out through two methods: end-to-end (e2e) training or with frozen ResNet-50 weights, with a focus on the end-to-end retraining, which result in higher overall performance. We assess classifier performance by comparing the sensitivity at 95% and 98% specificity on test datasets, utilizing various models for evaluation.

Our breast mammogram experiments address two classification tasks: breast density (A,B vs. C,D) and BI-RADS category (1,2 vs. 3,4,5), both utilizing the Imagenet pre-trained model based on SwAV. In the former case, density categories A and B serve as the positive class, while in the latter, BI-RADS categories 1 and 2 are considered the positive class. Fine-tuning is executed end-to-end as before, to evaluate metric improvements at 95% and 98% specificity. Lastly, our credit card fraud detection involves a straightforward Multi-Layer Perceptron (MLP) network. Sensitivities are evaluated at much higher specificities of 99% and 99.99%.

The implementation of AUCReshaping has shown notable improvements in sensitivity ranging from 2 to 40% across various test datasets. The extent of enhancement is contingent on factors such as the level of reshaping or boosting applied in the high-specificity region of the ROC curve and the baseline AUC scores. Importantly, these improvements exhibit a high degree of generalizability as they are demonstrated in the context of various diseases, domains, and with different pre-trained models. To further advance the state-of-the-art (SOTA) in terms of AUC scores and related metrics, it is possible to explore alternative architectures such as DenseNet or Vision-Transformer. By doing so, we can aim to achieve even better results and push the boundaries of performance in these classification tasks. It is worth noting that while boosting the output predictions can lead to substantial improvements, excessively high boosting values may perturb the output predictions to a degree that harms the overall network performance. As a result, identifying the optimal boosting value becomes a critical design consideration to maximize the performance benefits.

The AUCReshaping method offers a high degree of flexibility, as it can be applied at varying levels of granularity, depending on the specific requirements of the application. The selection of optimal thresholds and boosting values is highly dependent on the characteristics of the dataset and the particular design choices made. Indeed, the choice between retraining all layers during fine-tuning and fine-tuning only the linear classifier, has a significant impact on the AUC score improvement. Retraining all layers often results in higher AUC scores, but as the sensitivity at high-specificity for the baseline is lower in the latter case, a greater improvement is observed in the high-specificity sensitivities.

The choice of boosting strategy is also a crucial consideration. Whether to use a constant boosting value for all thresholds or weighted boosting for each threshold, can lead to different levels of improvement or changes in performance. It’s essential to strike a balance and avoid excessive boosting values, especially at thresholds far removed from the desired operating point, as this can lead to unexpected drops in performance. For e. g., if the improvement is desired at 98 or 99% specificity, applying the reshaping algorithm at the 60–80% range will most definitely cause the performance at 98% to drop. The distribution of the feature space within the manifold varies between different contrastive learning algorithms, resulting in similar AUC scores but distinct sensitivities at high specificities. The divergence in feature learning contributes to differences in output probabilities and decision boundaries among various models.

This research represents a significant step toward the ultimate goal of developing practical deep learning systems capable of optimizing binary classification tasks on heavily imbalanced datasets with significant misclassification costs. Ongoing research efforts may extend these ideas to combinations of multi-class classification tasks, while improving the global accuracy and explore approaches to address high-specificity misclassifications in the pre-training stage. The hope is that future research will continue to advance point-wise performance on ROC curves while also introducing practical metrics to assess the performance of such systems alongside AUC scores.

## Data Availability

The dataset utilized in this study comprised 2D X-ray imagery encompassing a total of 1,297,699 X-ray images, representing diverse anatomical regions such as the chest, spine, back, arm, leg, and others. The dataset was compiled from a combination of publicly available sources^25,27^ and proprietary, internal repositories^32^. Within this dataset, a subset of 16,693 chest X-ray images underwent meticulous annotation by a team of radiologists, encompassing the delineation of bounding boxes for pathologies spanning 16 distinct categories. For the purposes of this research, the 16k image subset was employed for the fine-tuning and assessment of the AUCReshaping technique. The study uses both data acquired from the resources that are publicly available as well as the data acquired for the project through collaboration and procurement agreements. The public data can be obtained using online resources and can be used under the applicable license guidance. Proper references are provided in the manuscript for the public data resources. The internal data acquired for the project can be available from the corresponding author upon a reasonable request.The Breast Mammogram dataset represents a substantial publicly available resource consisting of full-field digital mammography data^33,34^, which encompasses both BI-RADS assessments and annotations pertaining to abnormalities. This dataset comprises a total of 20,000 DICOM images, which correspond to 5000 distinct mammography examinations conducted between the years 2018 and 2020. The selection of these images was performed through random sampling, with a strict de-identification process being applied. Patient metadata, including details such as manufacturers and manufacturers’ model names, is included in the dataset, but notably, it excludes individuals aged 89 years or above. To ensure complete de-identification, patient-specific information present within the images is removed through predefined rectangular cropping. Furthermore, the de-identification process involves a meticulous review by two independent evaluators to ascertain the thorough removal of any patient-related data. The Breast mammogram dataset can be requested at https://github.com/vinbigdata-medical/vindr-mammo. The credit card dataset has been collected and analyzed through a research collaboration of Worldline and the Machine Learning Group^50^ of the Université Libre de Bruxelles (ULB) on big data mining and fraud detection. The credit card dataset can be downloaded from https://www.kaggle.com/datasets/mlg-ulb/creditcardfraud?resource=download.

## References

[1] Hassan, M. U., Rehmani, M. H. & Chen, J. Anomaly detection in blockchain networks: A comprehensive survey. In *IEEE Communications Surveys & Tutorials* (2022).

[2] Tang, Y.-X., Tang, Y.-B., Han, M., Xiao, J. & Summers, R. M. Abnormal chest x-ray identification with generative adversarial one-class classifier. In *2019 IEEE 16th International Symposium on Biomedical Imaging (ISBI 2019)*, 1358–1361 (IEEE, 2019).

[3] Shvetsova N, Bakker B, Fedulova I, Schulz H, Dylov DV (2021). Anomaly detection in medical imaging with deep perceptual autoencoders. IEEE Access.

[4] Bozorgtabar, B., Mahapatra, D., Vray, G. & Thiran, J.-P. Salad: Self-supervised aggregation learning for anomaly detection on x-rays. In *International Conference on Medical Image Computing and Computer-Assisted Intervention*, 468–478 (Springer, 2020).

[5] Bogdoll, D., Nitsche, M. & Zöllner, J. M. Anomaly detection in autonomous driving: A survey. In *Proceedings of the IEEE/CVF Conference on Computer Vision and Pattern Recognition*, 4488–4499 (2022).

[6] Jiang, X. et al. A survey of visual sensory anomaly detection. arXiv preprint arXiv:2202.07006 (2022).

[7] Hand DJ (2009). Measuring classifier performance: A coherent alternative to the area under the roc curve. Mach. Learn..

[8] Maniraj S, Saini A, Ahmed S, Sarkar S (2019). Credit card fraud detection using machine learning and data science. Int. J. Eng. Res..

[9] Ntalampiras S, Potamitis I, Fakotakis N (2011). Probabilistic novelty detection for acoustic surveillance under real-world conditions. IEEE Trans. Multimed..

[10] Kotsiantis S, Kanellopoulos D, Pintelas P (2006). Handling imbalanced datasets: A review. GESTS Int. Trans. Comput. Sci. Eng..

[11] Gu, Q., Zhu, L. & Cai, Z. Evaluation measures of the classification performance of imbalanced data sets. In *International Symposium on Intelligence Computation and Applications*, 461–471 (Springer, 2009).

[12] Chen, Z. et al. A comprehensive study on challenges in deploying deep learning based software. In *Proceedings of the 28th ACM Joint Meeting on European Software Engineering Conference and Symposium on the Foundations of Software Engineering*, 750–762 (2020).

[13] Baier, L., Jöhren, F. & Seebacher, S. Challenges in the deployment and operation of machine learning in practice. In ECIS, vol. 1 (2019).

[14] Danu, M. D. et al. Generation of radiology findings in chest x-ray by leveraging collaborative knowledge. arXiv preprint arXiv:2306.10448 (2023).

[15] Rudolph J (2022). Artificial intelligence in chest radiography reporting accuracy: Added clinical value in the emergency unit setting without 24/7 radiology coverage. Investig. Radiol..

[16] Hsiao, C.-Y., Lo, H.-Y., Yin, T.-C. & Lin, S.-D. Optimizing specificity under perfect sensitivity for medical data classification. In *2014 International Conference on Data Science and Advanced Analytics (DSAA)*, 163–169, 10.1109/DSAA.2014.7058068 (2014).

[17] Gündel S (2021). Robust classification from noisy labels: Integrating additional knowledge for chest radiography abnormality assessment. Med. Image Anal..

[18] Ghesu FC (2021). Quantifying and leveraging predictive uncertainty for medical image assessment. Med. Image Anal..

[19] Zhu, C., Mei, C., Jiang, H. & Zhou, R. Matrix-instance-based one-pass auc optimization. In *Pattern Recognition and Computer Vision: First Chinese Conference, PRCV 2018, Guangzhou, China*, Proceedings, Part III 1, 527–538 (Springer, 2018).

[20] Hu J, Yang H, Lyu MR, King I, So AM-C (2017). Online nonlinear AUC maximization for imbalanced data sets. IEEE Trans. Neural Netw. Learn. Syst..

[21] Gultekin S, Saha A, Ratnaparkhi A, Paisley J (2020). Mba: Mini-batch AUC optimization. IEEE Trans. Neural Netw. Learn. Syst..

[22] Doi K (2007). Computer-aided diagnosis in medical imaging: Historical review, current status and future potential. Comput. Med. Imaging Graph..

[23] Çallı E, Sogancioglu E, van Ginneken B, van Leeuwen KG, Murphy K (2021). Deep learning for chest x-ray analysis: A survey. Med. Image Anal..

[24] Chan H-P, Hadjiiski LM, Samala RK (2020). Computer-aided diagnosis in the era of deep learning. Med. Phys..

[25] Wang, X. et al. Chestx-ray8: Hospital-scale chest x-ray database and benchmarks on weakly-supervised classification and localization of common thorax diseases. In *Proceedings of the IEEE Conference on Computer Vision and Pattern Recognition*, 2097–2106 (2017).

[26] Irvin J (2019). Chexpert: A large chest radiograph dataset with uncertainty labels and expert comparison. Proc. AAAI Conf. Artif. Intell..

[27] Johnson AE (2019). Mimic-cxr, a de-identified publicly available database of chest radiographs with free-text reports. Sci. Data.

[28] Yalniz, I. Z., Jégou, H., Chen, K., Paluri, M. & Mahajan, D. Billion-scale semi-supervised learning for image classification. arXiv preprint arXiv:1905.00546 (2019).

[29] He, K., Fan, H., Wu, Y., Xie, S. & Girshick, R. Momentum contrast for unsupervised visual representation learning. In *Proceedings of the IEEE/CVF conference on computer vision and pattern recognition*, 9729–9738 (2020).

[30] Caron M (2020). Unsupervised learning of visual features by contrasting cluster assignments. Adv. Neural Inf. Process. Syst..

[31] Deng, J. et al. Imagenet: A large-scale hierarchical image database. In *2009 IEEE Conference on Computer Vision and Pattern Recognition*, 248–255 (IEEE, 2009).

[32] Ghesu FC (2022). Contrastive self-supervised learning from 100 million medical images with optional supervision. J. Med. Imaging.

[33] Nguyen HT (2023). Vindr-mammo: A large-scale benchmark dataset for computer-aided diagnosis in full-field digital mammography. Sci. Data.

[34] Goldberger AL (2000). Physiobank, physiotoolkit, and physionet: Components of a new research resource for complex physiologic signals. Circulation.

[35] Sickles, E. A., D’Orsi, C. J., Bassett, L. W. et al. ACR BI-RADS mammography. In *ACR BI-RADS Atlas, Breast Imaging Reporting and Data System*, 121–140 (American College of Radiology, Reston, VA, 2013).

[36] Kaiser, N. et al. Mammographic breast density classification using a deep neural network: assessment based on inter-observer variability. In *Medical Imaging 2019: Image Perception, Observer Performance, and Technology Assessment*, vol. 10952, 156–161 (SPIE, 2019).

[37] Lehman CD (2019). Mammographic breast density assessment using deep learning: Clinical implementation. Radiology.

[38] Carcillo F (2018). Scarff: A scalable framework for streaming credit card fraud detection with spark. Inf. Fusion.

[39] Lebichot, B., Le Borgne, Y.-A., He-Guelton, L., Oblé, F. & Bontempi, G. Deep-learning domain adaptation techniques for credit cards fraud detection. In *Recent Advances in Big Data and Deep Learning: Proceedings of the INNS Big Data and Deep Learning Conference INNSBDDL2019, held at Sestri Levante, Genova, Italy 16-18 April 2019*, 78–88 (Springer, 2020).

[40] Carcillo F (2021). Combining unsupervised and supervised learning in credit card fraud detection. Inf. Sci..

[41] Le Borgne Y-A, Bontempi G (2004). Machine learning for credit card fraud detection-practical handbook. ACM SIGKDD Explor. Newslett..

[42] Dal Pozzolo A, Boracchi G, Caelen O, Alippi C, Bontempi G (2017). Credit card fraud detection: A realistic modeling and a novel learning strategy. IEEE Trans. Neural Netw. Learn. Syst..

[43] Dal Pozzolo A, Caelen O, Le Borgne Y-A, Waterschoot S, Bontempi G (2014). Learned lessons in credit card fraud detection from a practitioner perspective. Expert Syst. Appl..

[44] Dal Pozzolo, A., Caelen, O., Johnson, R. A. & Bontempi, G. Calibrating probability with undersampling for unbalanced classification. In *2015 IEEE Symposium Series on Computational Intelligence*, 159–166 (IEEE, 2015).

[45] Dal Pozzolo A (2015). Adaptive machine learning for credit card fraud detection.

[46] Loshchilov, I. & Hutter, F. Decoupled weight decay regularization. In *International Conference on Learning Representations* (2018).

[47] Vickers AJ (2003). How many repeated measures in repeated measures designs? Statistical issues for comparative trials. BMC Med. Res. Methodol..

[48] Rueckel, J. et al. Pneumothorax detection in chest radiographs: Optimizing artificial intelligence system for accuracy and confounding bias reduction using in-image annotations in algorithm training. *Eur. Radiol*. 1–13 (2021). 10.1007/s00330-021-07833-w PMC8452588 33774722

[49] Chen, X., Xie, S. & He, K. An empirical study of training self-supervised vision transformers. In *Proceedings of the IEEE/CVF International Conference on Computer Vision*, 9640–9649 (2021).

[50] G. Bontempi, C. S. D. Machine learning group, université libre de bruxelles, brussels, belgium (2004). Accessed on March 2023.

